# Flexible Bayesian semiparametric mixed-effects model for skewed longitudinal data

**DOI:** 10.1186/s12874-024-02164-y

**Published:** 2024-03-01

**Authors:** Melkamu M. Ferede, Getachew A. Dagne, Samuel M. Mwalili, Workagegnehu H. Bilchut, Habtamu A. Engida, Simon M. Karanja

**Affiliations:** 1 Department of Statistics, University of Gondar, Gondar, Ethiopia; 2 Department of Epidemiology and Biostatistics, College of Public Health, University of South Florida, Tampa, FL 33612 USA; 3 Department of Statistics and Actuarial Sciences, Jomo Kenyatta University of Agriculture and Technology (JKUAT), Nairobi, Kenya; 4 Department of Internal Medicine, College of Medicine and Health Sciences, University of Gondar, Gondar, Ethiopia; 5 Department of Mathematics, Debre Markos University, Debre Markos, Ethiopia; 6 School of Public Health, Jomo Kenyatta University of Agriculture and Technology (JKUAT), Nairobi, Kenya

**Keywords:** Bayesian inference, Semiparametric mixed-models, Longitudinal data, Skew-distributions, Chronic kidney disease

## Abstract

**Background:**

In clinical trials and epidemiological research, mixed-effects models are commonly used to examine population-level and subject-specific trajectories of biomarkers over time. Despite their increasing popularity and application, the specification of these models necessitates a great deal of care when analysing longitudinal data with non-linear patterns and asymmetry. Parametric (linear) mixed-effect models may not capture these complexities flexibly and adequately. Additionally, assuming a Gaussian distribution for random effects and/or model errors may be overly restrictive, as it lacks robustness against deviations from symmetry.

**Methods:**

This paper presents a semiparametric mixed-effects model with flexible distributions for complex longitudinal data in the Bayesian paradigm. The non-linear time effect on the longitudinal response was modelled using a spline approach. The multivariate skew-t distribution, which is a more flexible distribution, is utilized to relax the normality assumptions associated with both random-effects and model errors.

**Results:**

To assess the effectiveness of the proposed methods in various model settings, simulation studies were conducted. We then applied these models on chronic kidney disease (CKD) data and assessed the relationship between covariates and estimated glomerular filtration rate (eGFR). First, we compared the proposed semiparametric partially linear mixed-effect (SPPLM) model with the fully parametric one (FPLM), and the results indicated that the SPPLM model outperformed the FPLM model. We then further compared four different SPPLM models, each assuming different distributions for the random effects and model errors. The model with a skew-t distribution exhibited a superior fit to the CKD data compared to the Gaussian model. The findings from the application revealed that hypertension, diabetes, and follow-up time had a substantial association with kidney function, specifically leading to a decrease in GFR estimates.

**Conclusions:**

The application and simulation studies have demonstrated that our work has made a significant contribution towards a more robust and adaptable methodology for modeling intricate longitudinal data. We achieved this by proposing a semiparametric Bayesian modeling approach with a spline smoothing function and a skew-t distribution.

**Supplementary Information:**

The online version contains supplementary material available at 10.1186/s12874-024-02164-y.

## Introduction

Longitudinal data are present in numerous clinical and other follow-up studies that involve monitoring subjects over time to understand the impact of exposures, processes, or characteristics on outcomes. These studies involve tracking a group of subjects and recording data at different time points throughout the study duration. For example, one or more renal functional progress biomarkers (e.g., serum creatinine, albuminuria, glomerular filtration rate, and other biomarkers) of a chronic kidney disease (CKD) patient can be measured repeatedly until end-stage renal disease and/or other events of interest occur.

This research was driven by longitudinal data on CKD, a significant global health issue that affects approximately 500 million individuals worldwide [1]. Around 80 percent of these CKD cases are found in low- and middle-income countries. A prevalence of approximately 35.52 percent of CKD was observed among people with diabetes in Ethiopia [2]. To comprehend how CKD progresses within individuals and across populations, as well as to assess the impact of treatments over time, conducting longitudinal data analysis is necessary.

Longitudinal data can show a variety of features over time and across subjects in many real-world situations during follow-up studies. A suitable choice of methods for analysing such complex longitudinal data is therefore sought. The most popular method proposed is the linear mixed-effects (LME) model [3–6] with a Gaussian response. The generalized linear mixed-effects models [7–9] and non-linear mixed-effects models [10] have been also used as an extension of LME model.

Despite the increasing popularity of LME models in applications, the specification and statistical inference of these models may necessitate much attention when treating and analysing longitudinal data with many features. One of these features is that longitudinal data can exhibit nonlinear, irregular patterns over time, along with asymmetry. Thus, to model and analyse longitudinal data with this feature, LME (fully parametric) models may not be flexible enough.

Another feature is that, unlike linear models, mixed models make assumptions regarding the distribution of model errors as well as random-effects. In the literature, it is usually assumed that the model errors and/or random-effects follow a multivariate normal distribution. In practice, longitudinal data might exhibit asymmetric distributions, leading to biased statistical results [11, 12]. Because of this, employing a normal distribution for model errors may lack robustness against deviations from normality and may be too limited to accurately describe the among- and within-subject variability of longitudinal outcomes [13]. Many previous studies suggest considering a more flexible distribution for model errors to make a valid statistical inference [13, 14]. There are different suggestions in the literature concerning the impact of misspecification of a random-effect distribution on parameter estimation and inference. For instance, Molenberghs and Verbeke [15] suggest that misspecification of the random-effects distribution can lead to biased parameter estimates in nonlinear and generalised linear mixed models; in linear mixed models, however, deviations from the normality assumption may have very little impact on parameter estimation. McCulloch and Neuhaus [16] considered a generalised linear mixed model using a maximum likelihood estimation technique to evaluate the misspecification of the distribution of a random effect. Their findings demonstrated the robustness of most aspects of statistical inferences to the normality of random effects. Other authors in the recent literature, however, suggest that future research should accept more flexible distributional assumption for random-effects in addition to model errors [17, 18]. As a result, skew distributions have recently been used in the literature to handle asymmetry and model longitudinal data more flexibly [18–20].

Thus, in this study, we propose a flexible Bayesian mixed-effects model in a semiparametric setting with a smoothing spline specification and skew distributions for longitudinal data with the aforementioned features. To assess the effectiveness of the proposed methods in various model specifications, simulation studies were conducted. Finally, the proposed model was applied to data on CKD.

## Methods

### Motivating CKD data and longitudinal outcome trajectories

This paper utilizes a dataset spanning eight years, from June 2014 to June 2022, in the context of chronic kidney disease (CKD). The CKD data was gathered from the University of Gondar Comprehensive Specialized Hospital in Ethiopia, primarily extracted from patients’ profiles (or charts) and medical records. Only patients with three or more follow-ups are included in the analysis. The dataset encompasses repeatedly recorded renal function biomarkers, comorbidities, and baseline characteristics of 198 CKD patients. On average, the patients were approximately 55 years old, with 56.6% being male. Around one-third (34.4%) of the CKD patients in the study population had baseline hypertension. Furthermore, the baseline prevalence of diabetes among the CKD patients was determined to be 23.81%.

**Fig. 1:**
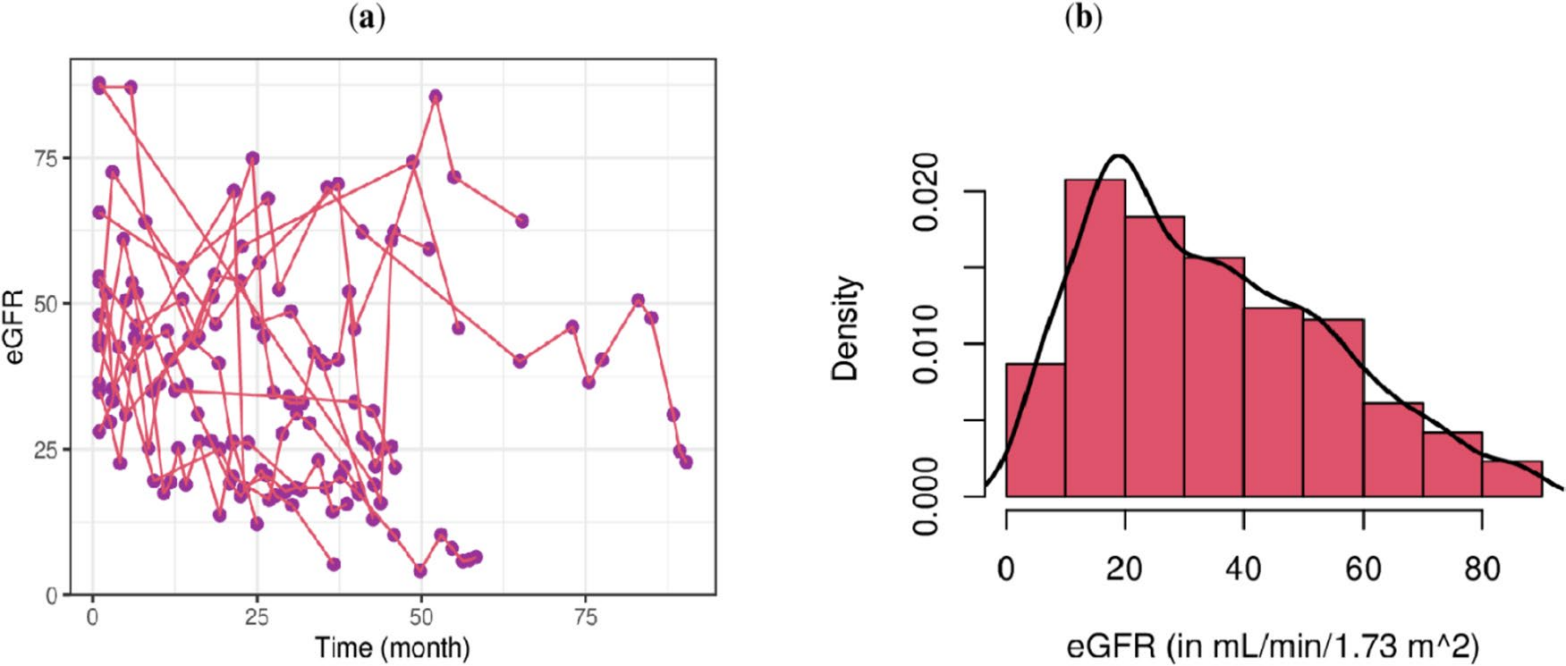
The trajectories and distribution of the outcome eGFR: (**a**) the line-plots of eGFR over time for some randomly selected patients, indicating non-linear patterns in the trajectories of eGFR; and (**b**) the histogram with density for all the patients, indicating that eGFR has a distribution that is skewed towards the left

The estimated glomerular filtration rate (eGFR), which estimates the rate at which the kidneys filter blood, is utilized as a longitudinal response variable. Thus, the analysis of this study considered 1,425 eGFR measurements from 189 patients. The minimum, maximum and average number of measurements per patient were 3, 18 and 8, respectively. 63.5% (120) patients had six and above number of measurements, and out of them 43% patients had ten and above measurements. Of the total 1,425 measurements, based on the National Kidney Foundation guidelines [21], 39.7% indicated CKD Stage 3 (moderate kidney disease), 32.9% indicated Stage 4 (severe kidney disease), and 14.6% indicated Stage 5 (end-stage renal disease). To accurately represent the diverse patterns of renal function progression and create an appropriate model, the analysis includes patients with an eGFR value below ninety. Figure 1 displays the eGFR profiles of patients with CKD. The figure depicts the presence of non-linear trajectories and a positively skewed distribution of eGFR over time.

### Bayesian modelling

#### The semiparametric mixed-effects longitudinal outcome model

In this paper, the longitudinal variable is denoted as ${y}_{ij}$, which represents the value of the response eGFR for subject $i$ at the ${j}^{th}$ time point ${t}_{ij}$. The indices $i$ and $j$ range from 1 to $m$ and 1 to ${m}_{i}$ respectively, indicating the total number of subjects and the number of measurements for each subject. Let ${{\varvec{x}}}_{ij}={({x}_{1ij}, \dots , {x}_{pij})}^{T}$ denotes a $1\times p$ vector of associated $p$ covariates. Most previous studies on chronic kidney disease have taken a parametric approach, like utilizing linear mixed-effects models, to model the longitudinal response variable ${y}_{ij}$ and the associated covariates ${{\varvec{x}}}_{ij}$. However, as demonstrated in the presentation of the motivating CKD data above, the outcome eGFR exhibits irregular (non-linear) trajectories over time. Therefore, this paper introduces a semiparametric mixed-effects model that considers the non-linear trajectories of ${{\varvec{y}}}_{i}$ by employing a spline approach.

1 $${{\varvec{y}}}_{i}={{\varvec{X}}}_{i}{\varvec{\beta}}+{{\varvec{N}}}_{i}\left({{\varvec{t}}}_{i}\right)+\boldsymbol{ }{{\varvec{H}}}_{i}{{\varvec{\xi}}}_{i}+{{\varvec{\varepsilon}}}_{i} , i=1, \dots , m,$$

where ${{\varvec{y}}}_{i}={({y}_{i1}, \dots , {y}_{imi})}^{T}$ represent the vector of longitudinal response variable, ${{\varvec{X}}}_{i}={({{\varvec{x}}}_{1i}, \dots , {{\varvec{x}}}_{pi})}^{T}$ denote the design matrix of fixed-effects, and ${{\varvec{H}}}_{i}={({{\varvec{h}}}_{1i}, \dots , {{\varvec{h}}}_{qi})}^{T}$ represent the design matrix of random-effects. ${\varvec{\beta}}$ and ${{\varvec{\xi}}}_{i}$ represent parameter vectors that are associated with the covariates of fixed and random effects. In Eq. (1), the effect of measurement time ${{\varvec{t}}}_{i}={({t}_{i1}, \dots , {t}_{imi})}^{T}$ on the response ${{\varvec{y}}}_{i}$ is modelled using a non-parametric approach. This is achieved by employing a smoothing function ${{\varvec{N}}}_{i}\left({{\varvec{t}}}_{i}\right)$, which can be defined as follows:

2 $${\mathbf{N}}_{i}\left({\mathbf{t}}_{i}\right)=f\left(\mathbf{U}\left({\mathbf{t}}_{i}\right),{\mathbf{V}}_{i}\left({\mathbf{t}}_{i}\right)\right)=\mathbf{U}\left({\mathbf{t}}_{i}\right)+{\mathbf{V}}_{i}\left({\mathbf{t}}_{i}\right),$$

where $\mathbf{U}\left({\mathbf{t}}_{i}\right)$ and ${\mathbf{V}}_{i}\left({\mathbf{t}}_{i}\right)$ represent unknown smoothing functions for the population and subject-specific variations of the longitudinal response ${{\varvec{y}}}_{i}$ due to time effects ${\mathbf{t}}_{i}$, respectively. The random vectors ${{\varvec{\xi}}}_{i}$, ${\mathbf{V}}_{i}\left({\mathbf{t}}_{i}\right),$ and ${{\varvec{\varepsilon}}}_{i}$ are assumed to be independent one another.

A regression spline method is utilized to specify the unknown functions $\mathbf{U}\left({\mathbf{t}}_{i}\right)$ and ${\mathbf{V}}_{i}\left({\mathbf{t}}_{i}\right)$ in Eq. (2), and can be defined as a linear combination of spline basis functions,

$${{\varvec{\Phi}}}_{ki}\left({\mathbf{t}}_{i}\right)={({{\varvec{\Phi}}}_{k} ({t}_{ij} ), \dots , {{\varvec{\Phi}}}_{k} ({t}_{imi}))}^{T}$$ and $${{\varvec{\Lambda}}}_{li}\left({\mathbf{t}}_{i}\right)={({{\varvec{\Lambda}}}_{l} ({t}_{ij} ), \dots , {{\varvec{\Lambda}}}_{l} ({t}_{imi} ))}^{T}$$. Where.

$${{\varvec{\Phi}}}_{k} \left({t}_{ij}\right)={({\upphi }_{0} ({t}_{ij} ),{\upphi }_{1} ({t}_{ij} ) \dots , {\upphi }_{k-1} ({t}_{ij} ))}^{T}$$ and.

${{\varvec{\Lambda}}}_{l} \left({t}_{ij}\right)={({\uplambda }_{0} ({t}_{ij} ),{\uplambda }_{1} ({t}_{ij} ) \dots , {\uplambda }_{l-1} ({t}_{ij} ))}^{T}; j=1, \dots ,{m}_{i}.$ Mathematically, the specification can be given by

3 $$\begin{array}{cc}\mathbf{U}\left({\mathbf{t}}_{i}\right)& \approx \sum\limits_{k=0}^{k-1}{{\varvec{\Phi}}}_{k}{\left({{\varvec{t}}}_{i}\right)}^{T}{\eta }_{k}={{\varvec{\Phi}}}_{k}\left({\mathbf{t}}_{i}\right){{\varvec{\eta}}}_{k} \\ {\mathbf{V}}_{i}\left({\mathbf{t}}_{i}\right)& \approx \sum\limits_{l=0}^{l-1}{{\varvec{\Lambda}}}_{l}{\left({{\varvec{t}}}_{i}\right)}^{T}{\vartheta }_{il}={{\varvec{\Lambda}}}_{l}\left({\mathbf{t}}_{i}\right){\boldsymbol{\vartheta }}_{il}\end{array}$$

where ${{\varvec{\eta}}}_{k}={({\upeta }_{0} ,{\upeta }_{1}, \dots , {\upeta }_{k-1} )}^{T}$ and ${\boldsymbol{\vartheta }}_{il}={({\vartheta }_{i0} ,{\mathrm{\vartheta }}_{i1}, \dots , {\mathrm{\vartheta }}_{i(l-1)} )}^{T}$ are $k\times 1$ and $l\times 1$ parameter vectors of the fixed-effect spline basis ${{\varvec{\Phi}}}_{k}\left({\mathbf{t}}_{i}\right)$ and random spline basis effects${{\varvec{\Lambda}}}_{l}\left({\mathbf{t}}_{i}\right)$, respectively. The B-spline, truncated power or natural cubic spline basis can be used to construct the bases (${{\varvec{\Phi}}}_{k}\left({\mathbf{t}}_{i}\right)$ and${{\varvec{\Lambda}}}_{l}\left({\mathbf{t}}_{i}\right)$) in (3). In this study, natural cubic spline with percentile-based knots is considered to approximate the bases. By using Eq. (3), model (1) can be rewritten as:

4 $${{\varvec{y}}}_{i}={{\varvec{X}}}_{i}{\varvec{\beta}}+{{\varvec{\Phi}}}_{k}\left({\mathbf{t}}_{i}\right){{\varvec{\eta}}}_{k}+\boldsymbol{ }{{\varvec{H}}}_{i}{{\varvec{\xi}}}_{i}+{{{\varvec{\Lambda}}}_{l}\left({\mathbf{t}}_{i}\right){\boldsymbol{\vartheta }}_{il}+\boldsymbol{ }{\varvec{\varepsilon}}}_{i} , i=1, \dots , m$$

Let ${{\varvec{Z}}}_{i}=({{\varvec{X}}}_{i}, {{\varvec{\Phi}}}_{k}\left({\mathbf{t}}_{i}\right))$ and ${{\varvec{R}}}_{i}=({{\varvec{H}}}_{i}, {{\varvec{\Lambda}}}_{l}\left({\mathbf{t}}_{i}\right))$ be the fixed-effect (population) and random effects design matrices, respectively. Furthermore, let $\boldsymbol{\alpha }={({{\varvec{\beta}}}_{p}^{T}, {{\varvec{\eta}}}_{k}^{T})}^{T}$ and ${\boldsymbol{\varphi }}_{i}={({{\varvec{\xi}}}_{iq}^{T}, {\boldsymbol{\vartheta }}_{il}^{T})}^{T}$ be the associated parameter vectors. Then, model (1) can be reformulated as

5 $$\begin{array}{cc}{\mathbf{y}}_{i}& ={\mathbf{Z}}_{i}\boldsymbol{\alpha }+{\mathbf{R}}_{i}{\boldsymbol{\varphi }}_{i}+{{\varvec{\varepsilon}}}_{i}, i=1,\dots ,m\\ {\boldsymbol{\varphi }}_{i}& \sim S{T}_{q+l,{\rho }_{\varphi }}\left(0,{{\varvec{\Sigma}}}_{\varphi },{{\varvec{\delta}}}_{\varphi }\right)\\ {{\varvec{\varepsilon}}}_{i}& \sim S{T}_{{m}_{i},{\rho }_{\varepsilon }}\left(0,{\sigma }_{\varepsilon }^{2}{\mathbf{I}}_{{m}_{i}},{\delta }_{\varepsilon }{\mathbf{I}}_{{m}_{i}}\right)\end{array}$$

Most previous studies assumed a Gaussian distribution for the random-effects ${\boldsymbol{\varphi }}_{i}$ (representing inter-subject variation of ${\mathbf{y}}_{i}$) as well as for the model errors ${{\varvec{\epsilon}}}_{i}$ (representing within-subject variation) due to its computational convenience. However, in this study, we considered multivariate skew-t distributions [22] for both ${\boldsymbol{\varphi }}_{i}$ and ${{\varvec{\varepsilon}}}_{i}$. That is, ${\boldsymbol{\varphi }}_{i}\sim S{T}_{q+l,{\rho }_{\varphi }}\left(0,{{\varvec{\Sigma}}}_{\varphi },{{\varvec{\delta}}}_{\varphi }\right)$ and ${{\varvec{\varepsilon}}}_{i}\sim S{T}_{{m}_{i},{\rho }_{\varepsilon }}\left(0,{\sigma }_{\varepsilon }^{2}{\mathbf{I}}_{{m}_{i}},{\delta }_{\varepsilon }{\mathbf{I}}_{{m}_{i}}\right)$. Where $ST(.)$ is a skew-t distribution; ${\rho }_{\varphi }$ and ${\rho }_{\varepsilon }$ denote degrees of freedom; ${{\varvec{\Sigma}}}_{\varphi }$ and ${{\varvec{\Sigma}}}_{\varepsilon }={\sigma }_{\varepsilon }^{2}{\mathbf{I}}_{{m}_{i}}$ denote covariance matrices; and ${{\varvec{\delta}}}_{\varphi }$ and ${{\varvec{\delta}}}_{\varepsilon }={\delta }_{\varepsilon }{\mathbf{I}}_{{m}_{i}}$ are skewness vectors of the random-effects ${\boldsymbol{\varphi }}_{i}$ and model errors ${{\varvec{\varepsilon}}}_{i}$, respectively.

#### Hierarchical reformulation of the model

The statistical inference from a semiparametric mixed-effects model with multivariate skew-t distributions using the likelihood approach can be computationally demanding. Hence, to overcome this challenge, we adopted the Bayesian approach, which offers computational efficiency. This approach not only reduces the computational load but also allows for more accurate parameter estimation by leveraging existing information (prior knowledge) for parameter estimation. By employing Markov Chain Monte Carlo (MCMC) algorithms, the Bayesian approach enables us to estimate the parameters more efficiently while obtaining posterior distributions that provide a comprehensive quantification of parameter uncertainty.

In order to carry out the MCMC, it is crucial to reformulate the model (5) by rep-resenting the skew-t distributions using the stochastic representation considered by [22] (See Appendix B). To achieve this, we introduced random vectors ${{\varvec{W}}}_{{\varphi }_{i}}={({W}_{{\varphi }_{i}1},\dots ,{W}_{{\varphi }_{i}(q+1)})}^{T}$ and ${{\varvec{W}}}_{{{\varvec{\varepsilon}}}_{i}}={({W}_{{\varepsilon }_{i}1},\dots ,{W}_{{\varepsilon }_{i}{m}_{i}})}^{T}$, as well as random variables ${v}_{\varphi }$ and ${v}_{\varepsilon }$ to represent the skew-t distributions associated with the random effects ${\boldsymbol{\varphi }}_{i}$ and model errors ${{\varvec{\varepsilon}}}_{i}$, respectively. Consequently, we present the hierarchical reformulation of model (5) as follows:

6 $$\begin{array}{c}{\mathbf{y}}_{i}|{\boldsymbol{\varphi }}_{i},{\mathbf{W}}_{\varepsilon i},{v}_{\varepsilon i};\boldsymbol{\alpha },{\sigma }_{\varepsilon }^{2},{{\varvec{\Sigma}}}_{\varphi },{\delta }_{\varepsilon },{\rho }_{\varepsilon }\sim {N}_{{m}_{i}}\left({\mathbf{Z}}_{i}\boldsymbol{\alpha }+{\mathbf{R}}_{i}{\boldsymbol{\varphi }}_{i}+{\delta }_{\varepsilon }{\mathbf{W}}_{\varepsilon i},{v}_{\varepsilon i}^{-1}{\sigma }_{\varepsilon }^{2}{1}_{{m}_{i}}\right),\\ {\varphi }_{i}|{\mathbf{W}}_{\varphi i},{v}_{\varphi i},{{\varvec{\Sigma}}}_{\varphi },{{\varvec{\delta}}}_{\varphi },{\rho }_{\varphi }\sim {N}_{q+l}\left({{\varvec{\delta}}}_{\varphi }{\mathbf{W}}_{\varphi i},{v}_{\varphi i}^{-1}{{\varvec{\Sigma}}}_{\varphi }\right),\\ {\mathbf{W}}_{\varphi i}|{v}_{\varphi i}\sim {N}_{q+l}\left(0,{v}_{\varphi i}^{-1}{\mathbf{I}}_{q+l}\right)I\left({\mathbf{W}}_{\varphi i}>0\right),\\ {\mathbf{W}}_{\varepsilon i}|{v}_{\varepsilon i}\sim {N}_{{m}_{i}}\left(0,{v}_{\varepsilon i}^{-1}{\mathbf{I}}_{{m}_{i}}\right)I\left({\mathbf{W}}_{\varepsilon i}>0\right),\\ {v}_{\varepsilon i}\left|{\rho }_{\epsilon }\sim\Gamma \left({\rho }_{\varepsilon }/2,{\rho }_{\varepsilon }/2\right), {v}_{\varphi i}\right|{\rho }_{\varphi }\sim\Gamma \left({\rho }_{\varphi }/2,{\rho }_{\varphi }/2\right)\end{array}$$

Where ${N}_{b}()$, in general, stands for a multivariate normal distribution with a dimension of b, and $\Gamma ()$ denotes a gamma distribution.

#### Prior specification and the posterior distribution

Let ${\varvec{\Omega}}=\left\{\boldsymbol{\alpha },{\sigma }_{\varepsilon }^{2},{{\varvec{\Sigma}}}_{\varphi }, {{\varvec{\delta}}}_{\varphi }, {\delta }_{\epsilon },{\rho }_{\varphi },{\rho }_{\varepsilon }\right\}$ represent the set of all parameters in the hierarchical model (6). We specify the prior distributions for each parameter in ${\varvec{\Omega}}$ as follows:

- The fixed-effects and skewness parameters $\boldsymbol{\alpha }$, ${{\varvec{\delta}}}_{\varphi }$, and ${\delta }_{\epsilon }$ are assumed to follow independent normal prior distributions ${N}_{{\text{p}}}({{\varvec{\upalpha}}}_{0}, {{\varvec{\Theta}}}_{\boldsymbol{\alpha }})$, ${N}_{{\text{q}}+{\text{l}}}(0, {{\varvec{\upkappa}}}_{{\delta }_{\varphi }})$, and $N(0, {\upkappa }_{{\delta }_{\varepsilon }})$, respectively.
- The scale parameters ${{\varvec{\Sigma}}}_{\varphi }$ and ${\sigma }_{\varepsilon }^{2}$ follow an inverse-Wishart and inverse-Gamma prior distributions, ${IW}_{{\text{q}}+{\text{l}}}({\mathbf{D}}_{\varphi }, {\upnu }_{\varphi })$ and $IG\left({\varrho }_{\varepsilon 1}, {\varrho }_{\varepsilon 2}\right),$ respectively.
- The degrees of freedom parameters ${\rho }_{\varphi }$ and ${\rho }_{\varepsilon }$ are assumed to follow truncated exponential prior distributions, $Exp({\rho }_{\vartheta 0})I({\rho }_{\vartheta }>3)$ and $Exp({\rho }_{\varepsilon 0})I({\rho }_{\varepsilon }>3)$, respectively.

The hyperparameter matrices ${{\varvec{\Theta}}}_{\boldsymbol{\alpha }}$ and ${\mathbf{D}}_{\varphi }$ are assumed diagonal for convenient implementation. Then, the prior distribution of all the parameters, denoted as $\pi ({\varvec{\Omega}})$, can be defined as the product of the individual prior distributions of each parameter.

Suppose $\mathbf{G}=\left\{{\mathbf{y}}_{i},{\mathbf{Z}}_{i}, {\mathbf{W}}_{{\varphi }_{i}}, {\mathbf{W}}_{{\varepsilon }_{i}},{v}_{\varphi }, {v}_{\varepsilon }\right\}$ be the observed data. An approximation of the posterior density of **Ω** given **G** can be obtained as follows:

7 $$\begin{array}{cc}\pi ({\varvec{\Omega}}|\mathcal{G})& \propto f(\mathcal{G}|{\varvec{\Omega}})\times \pi ({\varvec{\Omega}})\\ & \propto \prod\limits_{i=1}^{m}{\int }_{{\varphi }_{i}}\left\{{\left({\sigma }_{\epsilon }^{2}\right)}^{-\frac{{m}_{i}}{2}}{\text{exp}}\left(-\frac{1}{2}{\left({\mathbf{y}}_{i}-{{\varvec{\mu}}}_{y}\right)}^{T}{\left(\frac{{\sigma }_{\epsilon }^{2}{\mathbf{I}}_{{m}_{i}}}{{v}_{\epsilon i}}\right)}^{-1}\left({\mathbf{y}}_{i}-{{\varvec{\mu}}}_{y}\right)\right) \right\}\\ & \times {\left|{{\varvec{\Sigma}}}_{\varphi }\right|}^{-\frac{1}{2}}{\text{exp}}\left(-\frac{1}{2}{\left({\boldsymbol{\varphi }}_{i}-{{\varvec{\delta}}}_{\varphi }{\mathbf{W}}_{\varphi }\right)}^{T}{v}_{\varphi i}{{\varvec{\Sigma}}}_{\varphi }^{-1}\left({\boldsymbol{\varphi }}_{i}-{{\varvec{\delta}}}_{\varphi }{\mathbf{W}}_{\varphi i}\right)\right)\\ & \times {\text{exp}}\left(-\frac{1}{2}{v}_{\varphi i}{\mathbf{W}}_{\varphi i}^{T}{\mathbf{W}}_{\varphi i}\right)\times {\text{exp}}\left(-\frac{1}{2}{v}_{\epsilon i}{\mathbf{W}}_{\epsilon i}^{T}{\mathbf{W}}_{\epsilon i}\right)\\ & \left\{\frac{1}{\Gamma \left({\rho }_{\varphi }/2\right){\left({\rho }_{\varphi }/2\right)}^{{\rho }_{\varphi }/2}}{v}_{\varphi i}^{\frac{{\rho }_{\varphi }}{2}-1}\right\}{\text{exp}}\left(-\frac{2}{{\rho }_{\varphi }}{v}_{\varphi i}\right)\\ & \left.\left\{\frac{1}{\Gamma \left({\rho }_{\epsilon }/2\right){\left({\rho }_{\epsilon }/2\right)}^{{\rho }_{\epsilon }/2}}{v}_{\epsilon i}^{\frac{{\rho }_{\epsilon }}{2}}-1\right\}{\text{exp}}\left(-\frac{2}{{\rho }_{\epsilon }}{v}_{\epsilon i}\right)\right\}d{\varphi }_{i}\\ & \times {\text{exp}}\left(-\frac{1}{2}{\left(\boldsymbol{\alpha }-{\boldsymbol{\alpha }}_{0}\right)}^{T}{{\varvec{\Theta}}}_{\alpha }^{-1}\left(\boldsymbol{\alpha }-{\boldsymbol{\alpha }}_{0}\right)\right)\\ & \times {\left({\sigma }_{\epsilon }^{2}\right)}^{-{\varrho }_{\epsilon 1}-1}{\text{exp}}\left(-{\varrho }_{\epsilon 2}/{\sigma }_{\epsilon }^{2}\right)\\ & \times {\left|{{\varvec{\Sigma}}}_{\varphi }\right|}^{-\frac{\left({\nu }_{\varphi }+q+l+1\right)}{2}}{\text{exp}}\left(-\frac{1}{2}{\text{tr}}\left({\mathbf{D}}_{\varphi }{{\varvec{\Sigma}}}_{\varphi }^{-1}\right)\right)\\ & \times {\text{exp}}\left(-\frac{1}{2}{{\varvec{\delta}}}_{\varphi }^{T}{\left|{\kappa }_{{\delta }_{\varphi }}\right|}^{-1}{{\varvec{\delta}}}_{\varphi }\right)\times {\text{exp}}\left(-\frac{1}{2{\kappa }_{{\delta }_{\epsilon }}}{\delta }_{\epsilon }^{2}\right)\\ & \times {\text{exp}}\left(-{\rho }_{\varphi }{\rho }_{\varphi }\right)\times {\text{exp}}\left(-{\rho }_{\epsilon 0}{\rho }_{\epsilon }\right)\end{array}$$

where $f(\mathcal{G}|{\varvec{\Omega}})$ is the joint likelihood function of **G** given **Ω** and ${{\varvec{\mu}}}_{y}={\mathbf{Z}}_{i}\boldsymbol{\alpha }+{\mathbf{R}}_{i}{\boldsymbol{\varphi }}_{i}+{\delta }_{\varepsilon }{\mathbf{W}}_{\varepsilon i}$.

The Metropolis–Hastings algorithm within Gibbs sampler can be used to draw samples from the full conditional posterior distributions of the parameters and to estimate their posterior means and standard deviations. For all models, the Markov chain Monte Carlo (MCMC) procedure was implemented using WinBUGS14 software, which simplifies the implementation of the MCMC algorithm by eliminating the need to derive full conditionals and specify the algorithm explicitly.

#### Model comparison and diagnostics checking

The specification and implementation of the proposed model in the Bayesian approach may require to conduct convergence diagnostic checks and thoroughly examine the distributional assumptions before drawing any statistical inferences about the parameters. Failure to do so may result in biased estimates and invalid statistical inference. Thus, in this study, the Brooks-Gelman-Rubin (BGR) plot [23], trace plot, ACF plot and the Geweke’s test of convergence are all used to evaluate convergence. After confirming convergence, we proceed to evaluate the effectiveness of the proposed semiparametric mixed-effects model (5) by exploring various distributional assumptions for the random-effects and model errors. This model comparison involves considering different distributional specifications and examining their performance in capturing the underlying characteristics of the data. These specifications are given below:

- **MoSTST**: A semiparametric partially linear mixed-effects model (SPPLMEM) with multivariate skew-t (ST) distributions for both the random- effects ${\boldsymbol{\varphi }}_{i}$ and model errors ${{\varvec{\varepsilon}}}_{i}$.
- **MoNST**: An SPPLMEM with multivariate normal (N) distribution of ${\boldsymbol{\varphi }}_{i}$ and ST-distribution of ${{\varvec{\varepsilon}}}_{i}$.
- **MoSNSN**: An SPPLMEM with both ${\boldsymbol{\varphi }}_{i}$ and ${{\varvec{\varepsilon}}}_{i}$ follow multivariate skew-normal (SN) distributions.
- **MoNN**: An SPPLMEM with multivariate normal (N) distributions of ${\boldsymbol{\varphi }}_{i}$ and ${{\varvec{\varepsilon}}}_{i}$. MoNN model is the standard choice in longitudinal data analysis.

In order to evaluate the performance of the estimators and make comparisons between different models, we utilised several statistical measures. Additionally, to compare and select the best-fitting Bayesian model for the skewed longitudinal response, we employed the deviance information criterion, which takes into account both the goodness of fit and model complexity.

#### Deviance information criterion (DIC)

In this paper, DIC [24] is used to choose the best-fitting Bayesian semiparametric mixed-effects model. DIC is the most popular Bayesian model comparison tool in the literature: the smaller this value, the better the model fit. The DIC for the hierarchical Bayesian model (6) with parameters vector Ω and observed longitudinal data D can be defined as

8 $$DIC=Dev\left(\overline{\Omega }\right)+2{\mathcal{P}}_{\mathcal{D}}$$

where

9 $$Dev\left(\overline{\Omega }\right)={\text{Dev}}\left(E\left(\Omega |{\varvec{D}}\right)\right)$$

is the deviance computed at the posterior mean of model parameters. And

10 $${\mathcal{P}}_{\mathcal{D}}={\overline{Dev(\Omega)}}-Dev\left(\overline{\Omega }\right)$$

is effective number of parameters. Where $\stackrel{-}{Dev(\Omega )}$ represents the expected deviance; $Dev\left(\Omega \right)=-2{\text{log}}(f({\varvec{D}}|{\varvec{\Omega}}))$ is the deviance function; and $f({\varvec{D}}|{\varvec{\Omega}})$ is the likelihood of the parameters in Eq. (6).

## Results

### Simulation studies

Simulation studies were conducted to assess and compare the effectiveness of the proposed semiparametric mixed-effects model in various model settings. In these simulations, a sample of 400 individuals was considered, each having eleven equally spaced measurement times, resulting in a total of 4,400 observations. The longitudinal data was simulated using the semiparametric mixed-effects model (5). The specifications of this general model can be given as follows:

11 $$\begin{array}{cc}{y}_{ij}& ={\alpha }_{1}+{\alpha }_{2}*{Z}_{1ij}+{\alpha }_{3}*{Z}_{2ij}+{\varphi }_{i1}+\left({\lambda }_{1}+{\varphi }_{i2}\right)*{\phi }_{1}\left({t}_{ij}\right)\\ & +\left({\lambda }_{2}+{\varphi }_{i3}\right)*{\phi }_{2}\left({t}_{ij}\right)+\left({\lambda }_{3}+{\varphi }_{i4}\right)*{\phi }_{3}\left({t}_{ij}\right)+{\varepsilon }_{ij}\end{array}$$

where ${y}_{ij}$ and ${Z}_{pij}$ are the longitudinal response and binary covariates, $p=1, 2$. $\boldsymbol{\alpha }={({\alpha }_{1}, {\alpha }_{2}, {\alpha }_{3})}^{T}$ and ${\varvec{\lambda}}={({\lambda }_{1}, {\lambda }_{2}, {\lambda }_{3})}^{T}$ denote parameter vectors of the fixed effects and ${\boldsymbol{\varphi }}_{i}=$ (${\varphi }_{i1}$, ${\varphi }_{i2}$, ${\varphi }_{i3}$, ${\varphi }_{i4}$)^T^ denote parameter vector of the random-effects. ${\varvec{\Phi}}\left({t}_{ij}\right)=$ (${\phi }_{1}\left({t}_{ij}\right)$, ${\phi }_{2}\left({t}_{ij}\right)$,${\phi }_{3}\left({t}_{ij}\right)$)^T^ is a vector of natural cubic spline bases used in the regression spline method. We used eleven equally spaced time points (t_ij_ = 0, 1, 2, 3, …, 10) with percentile based knots to generate the spline bases [14, 25, 26].

To create longitudinal data with a skewed distribution, the components of the random effects ${\boldsymbol{\varphi }}_{i}$ and the error terms ${{\varvec{\varepsilon}}}_{i}$ are simulated from a gamma distribution $\gamma (\mathrm{2,1})$. These generated values are then subtracted by two [27, 28]. The vectors **α** = (27.5, − 5, − 4)^T^ and **λ** = (− 9, − 25, − 5) are set accordingly.

Furthermore, ${{\varvec{Z}}}_{1}$ and ${{\varvec{Z}}}_{2}$ are generated using Bernoulli distributions with probabilities (proportions) 0.24 and 0.44, respectively.

While performing the Bayesian inference, we considered weakly informative priors for the parameters. Specifically, each component of $\boldsymbol{\alpha },{\varvec{\uplambda}}, {{\varvec{\delta}}}_{\varphi },$ and ${\delta }_{\varepsilon }$ was assumed to follow a normal prior distribution, N (0, 100). Furthermore, inverse Wishart $IW(0.01{\mathbf{I}}_{4}, 4)$, inverse gamma *IG*(0.01, 0.01), *Exp*(0.5), and *Exp*(0.5) priors are considered for ${{\varvec{\Sigma}}}_{\varphi }$, ${\sigma }_{\varepsilon }^{2}$, ${\rho }_{\varphi }$, and ${\rho }_{\varepsilon }$, respectively.

Three MCMC chains were run using R2WinBUGS in R. Each chain consisted of 90,000 iterations, and a burn-in of 45,000 iterations was applied. After thinning, we retained a total of 4,500 posterior estimates for each parameter from each model.

In assessing convergence, Figure A.1 (Appendix A) displays the trace plots, while Figure A.2 (Appendix A) exhibits the plots of ACF (autocorrelation function) and BGR diagnostic plots of the parameters derived from the proposed semiparameteric mixed-effects model [5). These figures clearly demonstrate convergence. In addition, none of the absolute values of Geweke's test statistics results (Appendix A) for the parameters exceeded the 95% critical value of 1.96, demonstrating strong evidence of convergence.

We computed the relative bias (RB), which indicates the extent of bias in the estimators; the 95% coverage probability (CP) to assess the accuracy of credible intervals; and the root-mean-square (RMS) error to measure the overall prediction accuracy. The results presented in Table 1 provide an evaluation of different models in terms of their posterior mean estimates, along with RB, RMSE, CP, and DIC based on simulation studies. Specifically, the evaluation focuses on semiparametric mixed-effect models (SPMEMs) with skew distributions in comparison to a Gaussian SPME model for skewed longitudinal data. The findings indicate that the proposed Bayesian SPMEMs with skew distributions (MoSTST, MoNST, and MoSNSN) outperformed the Gaussian model (MoNN). The DIC values of the skewed models MoNST, MoSNSN, and MoSTST are comparatively smaller (11,674, 12,997, and 13,249, respectively) than those of the normal model MoNN (DIC = 14,528). The two models with skew-t and skew-normal distributions of model errors and random effects (MoSTST and MoSNSN) have relatively closer DIC values. Specifically, the model with a skew-t distribution for model errors and a normal distribution of random effects (MoNST) has the smallest DIC value and exhibited better performance compared to the other models. In terms of relative bias (RB) and RMSE, however, the model with a skew-t distribution for both random effects and model errors (MoSTST) demonstrated superior performance. This suggests that incorporating skewness in modelling the longitudinal data and proposing a more flexible distributional assumption (skew distribution) allows for better capturing the inherent asymmetries and heavy tails present in the data, leading to more accurate estimates. Overall, these results emphasize the advantages of employing Bayesian SPMEM with skew distribution over the conventional Gaussian model, offering greater flexibility and improved performance in accurately modelling complex longitudinal data.

**Table 1:** Simulation Results: Parameter Estimates (Est) with True Value (TV), RB, RMS Error, CP, and DIC for Each Model

Par	TV	Method	Models			
			MoSTST	MoNST	MoSNSN	MoNN
${\alpha }_{1}$	27.5	Est	27.505	27.506	27.456	29.981
		RB	0.000	0.036	-0.002	0.090
		RMS	0.123	0.502	0.564	1.984
		${\text{CP}}$	94.76	94.91	93.71	79.34
${\alpha }_{2}$	-5.0	Est	-4.870	-4.867	-4.891	-4.874
		RB	-0.026	-0.027	-0.022	-0.025
		RMS	0.155	0.159	0.134	0.160
		${\text{CP}}$	67.27	66.80	72.09	53.44
${\alpha }_{3}$	-4.0	Est	-3.945	-3.919	-3.939	-3.845
		RB	-0.014	-0.020	-0.015	-0.039
		RMS	0.085	0.114	0.088	0.175
		${\text{CP}}$	86.47	87.13	84.18	85.24
${\lambda }_{1}$	-9.0	Est	-8.933	-8.923	-8.972	-8.851
		RB	-0.007	-0.009	-0.003	-0.017
		RMS	0.399	0.198	0.439	0.225
		CP	95.24	95.00	95.89	87.93
${\lambda }_{2}$	-25.0	Est	-25.520	-25.454	-25.566	-25.208
		${\text{RB}}$	0.021	0.018	0.023	0.008
		RMS	0.605	0.541	0.653	0.348
		CP	60.04	62.96	59.60	87.93
${\lambda }_{3}$	-5.0	Est	-5.056	-4.960	-5.047	-4.856
		RB	0.011	-0.008	0.010	-0.029
		RMS	0.521	0.140	0.561	0.193
		CP	96.62	94.96	97.58	79.38
${\sigma }_{\epsilon }^{2}$	0.5	Est	0.471	0.487	0.458	4.783
		RB	0.058	0.026	0.084	8.565
		RMS	0.07	0.064	0.077	4.285
		CP	92.69	94.93	90.64	32.51
${\sigma }_{{\varphi }_{1}}^{2}$	0.1	Est	0.087	0.113	0.103	0.197
		RB	-0.131	0.130	0.025	0.969
		RMS	0.035	0.061	0.038	0.145
		CP	96.53	94.49	95.40	86.29
${\sigma }_{{\varphi }_{2}}^{2}$	0.3	Est	0.329	0.397	0.367	0.499
		RB	0.097	0.323	0.222	0.662
		RMS	0.172	0.237	0.209	0.378
		CP	94.29	91.29	92.96	90.36
${\sigma }_{{\varphi }_{3}}^{2}$	0.3	Est	0.29	0.391	0.321	0.67
		RB	* − *0*.*032	0.304	0.07	1.232
		RMS	0.178	0.324	0.189	0.655
		CP	95.96	94.56	94.42	89.33
${\sigma }_{{\varphi }_{4}}^{2}$	0.4	Est	0.4	0.662	0.476	0.815
		RB	0	0.655	0.19	1.038
		RMS	0.17	0.413	0.241	0.549
		CP	95.64	87.47	92.91	80.22
DIC			13,249.70	11,674.80	12,997.80	14,528.40

### Results of the CKD data analysis

In this paper, we included diabetes and hypertension as binary covariates based on the real CKD dataset and three spline basis functions of time with four random-effects to model and analyse the longitudinal response, the estimated glomerular filtration rate (eGFR). Accordingly, we reformulate the general semiparametric mixed-effects model (6) as follows:

12 $$\begin{array}{cc}{eGFR}_{ij}& ={\alpha }_{1}+{\alpha }_{2}*{Diabetes}_{ij}+{\alpha }_{3}*{Hypertension}_{ij}+{\varphi }_{i1}+\left({\lambda }_{1}+{\varphi }_{i2}\right)*{\phi }_{1}\left({Time}_{ij}\right)\\ & +\left({\lambda }_{2}+{\varphi }_{i3}\right)*{\phi }_{2}\left({Time}_{ij}\right)+\left({\lambda }_{3}+{\varphi }_{i4}\right)*{\phi }_{3}\left({Time}_{ij}\right)+{\varepsilon }_{ij}\end{array}$$

where the parameter vectors **α**, **λ**, ${\boldsymbol{\varphi }}_{i}$, and **Φ**(${Time}_{ij}$) are as defined as in the simulation section study. In order to obtain an approximation of the spline bases, we considered two internal knots at 9 and 25 months and two boundary knots at 0 and 96 months. The locations of these knots were determined based on the quantiles of the distribution of observed measurement time points. We proceed to analyse the CKD data using the proposed model with varying distributional assumptions, and subsequently compare and interpret the results. We begin by initially comparing the performance of two models: the proposed semiparametric (partially linear) mixed-effects model (SPPLMEM) specified in Eq. (12), and a fully parametric (linear) mixed-effects model (FPLMEM) that assumes Gaussian distributions for both the random effects and model errors. The FPLMEM is specifically defined as follows:

13 $${eGFR}_{ij}={\alpha }_{1}+{\alpha }_{2}*{Diabetes}_{ij}+{\alpha }_{3}*{Hypertension}_{ij}+{b}_{i1}+\left({\alpha }_{4}+{b}_{i2}\right)*{Time}_{ij}+{\varepsilon }_{ij}$$

where ${Time}_{ij}$ denotes the observed measurement time of the longitudinal biomarkers for the i^th^ subject at the j^th^ visit. The results (Table 2) show that the estimates of some parameters become large from FPLMEM compared to SPPLMEM. For instance, the estimates of ${\alpha }_{2}$ and σ^2^ from SPPLMEM were − 6.38 and 60.19, while from FPLMEM they became − 7.58 and 72.30, respectively. In addition, in order to select the most suitable Bayesian model that accurately represents the CKD data, we also compute the deviance information criterion (DIC) [24]. Our analysis reveals that the SPPLMEM gives a lower DIC value (DIC = 10, 290) in comparison to the FPLMEM (DIC = 10, 430).

**Table 2:** Comparison of Parameter Estimates (PE) between the Proposed Semiparametric Mixed-Effects Model (SPPLMEM) and the Fully Parametric Mixed-Effects Model (FPLMEM)

Model	SPPLMEM			FPLMEM		
Par	PE	StD	Cl	PE	StD	Cl
${\alpha }_{1}$	55.29	1.58	(52*.*03*,* 58*.*09)	55.86	1.722	(52*.*28*,* 58*.*00)
${\alpha }_{2}$	* − *6*.*38	2.36	(*− *11*.*01*, − *1*.*80)	* − *7*.*58	2.17	(*− *11*.*94*, − *3*.*40)
${\alpha }_{3}$	* − *5*.*69	0.67	(*− *7*.*00*, − *4*.*32)	* − *6*.*25	0.67	(*− *7*.*60*, − *4*.*90)
${\sigma }_{\epsilon }^{2}$	60.19	3.03	(54*.*74*,* 66*.*61)	72.3	3.25	(66*.*14*,* 79*.*14)
DIC	10,290			10,430		

*StD* Standard Deviation*, CI* 95% Credible Interval

After selecting the SPPLMEM as the most suitable model that accurately represents the data, we proceed to further compare four different SPPLMEMs by taking into account different distributional specifications. For model errors and random-effects as described in the simulation study. We fitted four Bayesian semiparametric mixed-effects models to the CKD data. The MCMC setup, computations, and convergence diagnostic methods employed were identical to those described in the simulation study. Table 3. displays a summary of the data analysis results and estimates for the parameters (Par) obtained from the four models with different distributional specifications.

**Table 3:**
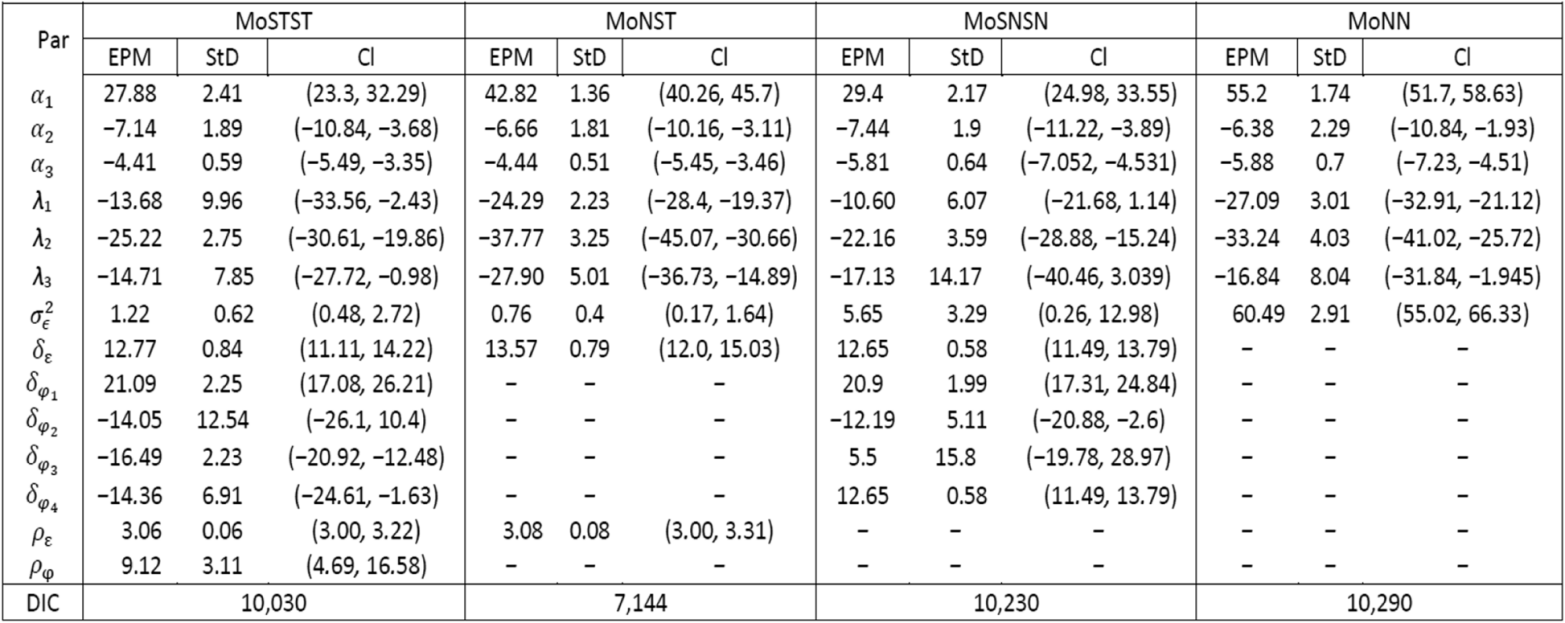
Summary results of CKD data analysis based on four Bayesian models with different distributional specifications

*EPM  *Estimated Posterior Mean*,  StD  *Standard Deviation*,  CI  *95% Credible Interval

As shown in Table 3., the CKD data analysis results reveal that each model produces slightly varied yet statistically significant estimates of most of the parameters. When comparing the models, the findings reveal that the utilization of the 4th model (MoNN), which employs a multivariate normal distribution for random effects and model errors, may result in an overestimation of some of the parameters. Specifically, the population parameters${\alpha }_{1}$, ${\alpha }_{2}$, ${\alpha }_{3}$, ${\lambda }_{1}$, ${\lambda }_{2}$, and ${\lambda }_{3}$ are prone to being overestimated. Notably, as can be clearly seen, the estimated scale parameter (the variance) of model errors (${\sigma }_{\varepsilon }^{2}$) is significantly larger in MoNN compared to the other models. The 3rd model (MoSNSN) also gives larger parameter estimates (e.g.,${\widehat{\sigma }}_{\varepsilon }^{2}$) compared to the first two models. Furthermore, the estimated skewness parameter of the outcome eGFR (${\delta }_{\varepsilon }$) is significantly different from zero in the first three models: MoSTST, MoNST, and MoSNSN. Some of the skewness parameters of the random effects (${{\varvec{\delta}}}_{\varphi }$) are also significantly different from zero in MoSTST and MoSNSN. Thus, the significantly different from zero positive estimates of ${\delta }_{\varepsilon }$ and the subject-specific random intercept ${\delta }_{{\varphi }_{1}}$ confirm the presence of positive skewness in the longitudinal eGFR data. In other words, the non-zero estimates of the skewness parameters and relatively small estimates of the variances may indicate that the proposed Bayesian models with skew-t distribution of model errors and/or random effects (MoSTST and MoNST) fit the CKD data well. This is in line with the results of the simulation studies.

In general, the proposed models (MoSTST and MoNST) outperform and the standard MoNN. In particular, MoNST has been chosen as the best model for further in-depth interpretation and discussion of the results because it has a relatively small DIC value, despite the fact that both MoSTST and MoSNSN have some significant skewness parameter estimates for the random effects. As can be seen from the simulation studies, MoNST also has a lower DIC value. This finding, a mixed model (skewed in our case) with a normal distribution of random-effects, is consistent with the study [15].

The results of all models indicate that the variables examined in this study, namely hypertension, diabetes, and follow-up time (the spline bases), are statistically significant factors contributing to the decline of patients’ kidney function. This is attributed to the negative and significant association between these covariates and the response variable, eGFR. In other words, it is evident that these covariates have a substantial association with the decrease in GFR estimates. For example, the diabetes coefficient (${\widehat{\alpha }}_{2}=-6.66$, 95% CI: [− 10.16, − 3.11]) from MoNST (the best-fitting model) can be interpreted as the eGFR value of a CKD patient with diabetes being reduced by 6.66 units compared to a CKD patient without diabetes, while holding the same covariates and random effects. Additionally, a hypertensive CKD patient is associated with a 4.44 unit lower eGFR value (${\widehat{\alpha }}_{3}=-4.44$, 95% CI: [− 5.45, − 3.46]) compared to a non-hypertensive CKD patient, with the same covariates and random effects.

## Discussion

In recent years, there has been a growing emphasis in the literature on effectively modeling longitudinal data with many features. This includes giving careful consideration to the functional forms of longitudinal markers and the assumptions made about the distribution of random effects and model errors. With this in mind, the main objective of this study was to develop a flexible Bayesian mixed-effects model that addresses the problems commonly observed in longitudinal CKD data, encompassing characteristics such as skewness, non-linear effects over time, and flexible distributions for both random effects and model errors. The ultimate goal was to establish a robust statistical methodology that enables accurate and reliable inference in complex longitudinal data analysis.

We therefore proposed a Bayesian semiparametric mixed-effects model for the longitudinal response eGFR that addresses the above issues. To capture the non-linear effects of time and the flexibility of eGFR, regression splines were employed in the model. Additionally, multivariate skew distributions were incorporated to account for skewness in eGFR and to relax the assumptions about its distribution. Simulation studies were first conducted to provide a comprehensive description and evaluation of the performance of the proposed model.

We applied the proposed model by analysing data on chronic kidney disease (CKD) and assessing the relationship between covariates and estimated glomerular filtration rate (eGFR). The model comparison process in this study involved two steps. Firstly, we compared the proposed semiparametric partially linear mixed-effect (SPPLM) model with the fully parametric one (FPLM), and our results indicated that the SPPLM model outperformed the FPLM model. In the second step, we further compared four different SPPLM models, each assuming different distributions for the random-effects and model errors. As described in the data analysis and results, the SPPLM models with skew-t distribution exhibited a superior fit to the CKD data in comparison to the Gaussian SPPLM model.

The findings from the application revealed that hypertension, diabetes, and follow-up time had a substantial association with kidney function, specifically leading to a decrease in eGFR. These factors were identified as important predictors and exhibited a negative correlation with kidney function.

Additionally, the results of this study imply that when dealing with longitudinal data characterized by the aforementioned features, it is useful to incorporate non-parametric smoothing functions (splines) to capture non-linear time-effects and utilize skew distributions for model errors and/or random effects. In particular, accounting for skewness in the longitudinal data analysis by utilizing a more flexible distribution, the skew-t distribution, is crucial to handle asymmetry in the data and get unbiased results. By doing so, we can obtain less biased results and draw valid statistical inferences. Additionally, employing a flexible distributional assumption for the random-effects can lead to a more accurate explanation of subject-specific variations.

Apart from the CKD follow-up data that served as motivation, our methodology has broader applicability in cases where the longitudinal data have similar characteristics and the fundamental model requirements (or settings) are satisfied.

## Conclusion

In conclusion, we have proposed a semiparametric Bayesian modeling approach with flexible distributions for complex longitudinal data. The results of the simulation and application studies have demonstrated that our work has made a significant contribution towards a more robust and adaptable methodology for modeling intricate longitudinal data. We recommend paying special attention to the specifications of the functional forms of longitudinal biomarkers and distributional assumptions of model errors when modeling complex longitudinal data.

### Supplementary Information

**Additional file 1:** **Appendix A.** Convergence diagnostic checking results. **Figure A.1.** Trace plots of some representative parameters from the chosen model. **Figure A.2.** Autocorrelation function plots (a) and BGR plots (b) of some representative parameters. **Table A.1.** Results of the Geweke's test of convergence. The computed value of the test statistic for each parameter from the chosen model. **Appendix B.** Skew Distributions.

## Data Availability

The actual CKD data utilized to exemplify the proposed model can be obtained from the corresponding author upon a substantial request.
