# Supplementary material for: Flexible Bayesian semiparametric mixed-effects model for skewed longitudinal data
Source: BMC Med Res Methodol. 2024 Mar 1;24:56. doi: 10.1186/s12874-024-02164-y (PMC10908071; doi:10.1186/s12874-024-02164-y)
Supplement: Supplementary file 1 — Additional file 1: Appendix A. Convergence diagnostic checking results. Figure A.1. Trace plots of some representative parameters from the chosen model. Figure A.2. Autocorrelation function plots (a) and BGR plots (b) of some representative parameters. Table A.1. Results of the Geweke's test of convergence. The computed value of the test statistic for each parameter from the chosen model. Appendix B. Skew Distributions. [file 12874_2024_2164_MOESM1_ESM.docx]

**Appendix A. Convergence diagnostic checking results**

In this subsection, the BGR plots, trace plots, ACF plots and Geweke's test results are presented.


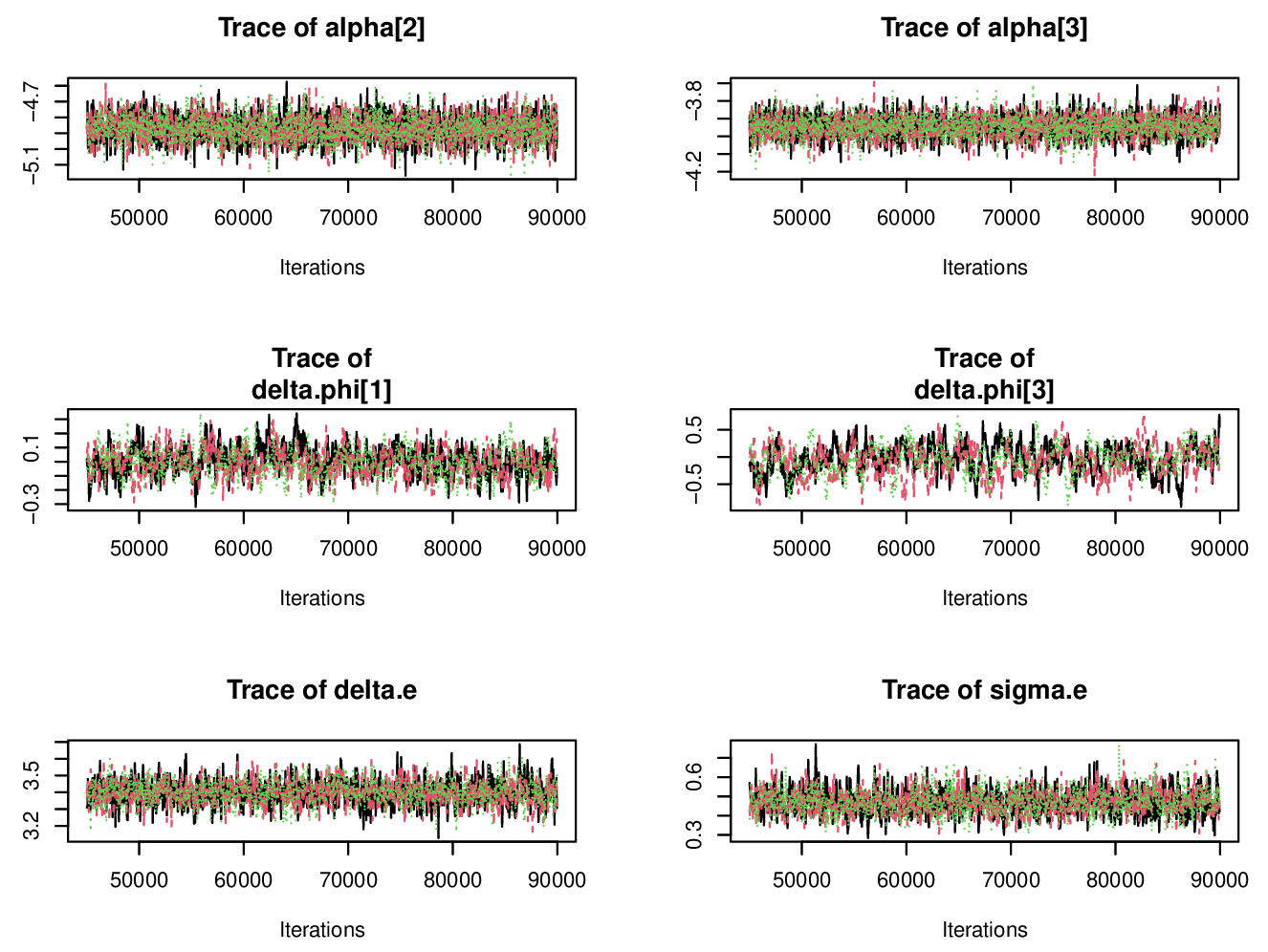


Figure A.1: Trace plots of some representative parameters from the chosen model. The plots depict well-mixing of each parameter’s three MCMC chains, indicating convergence.


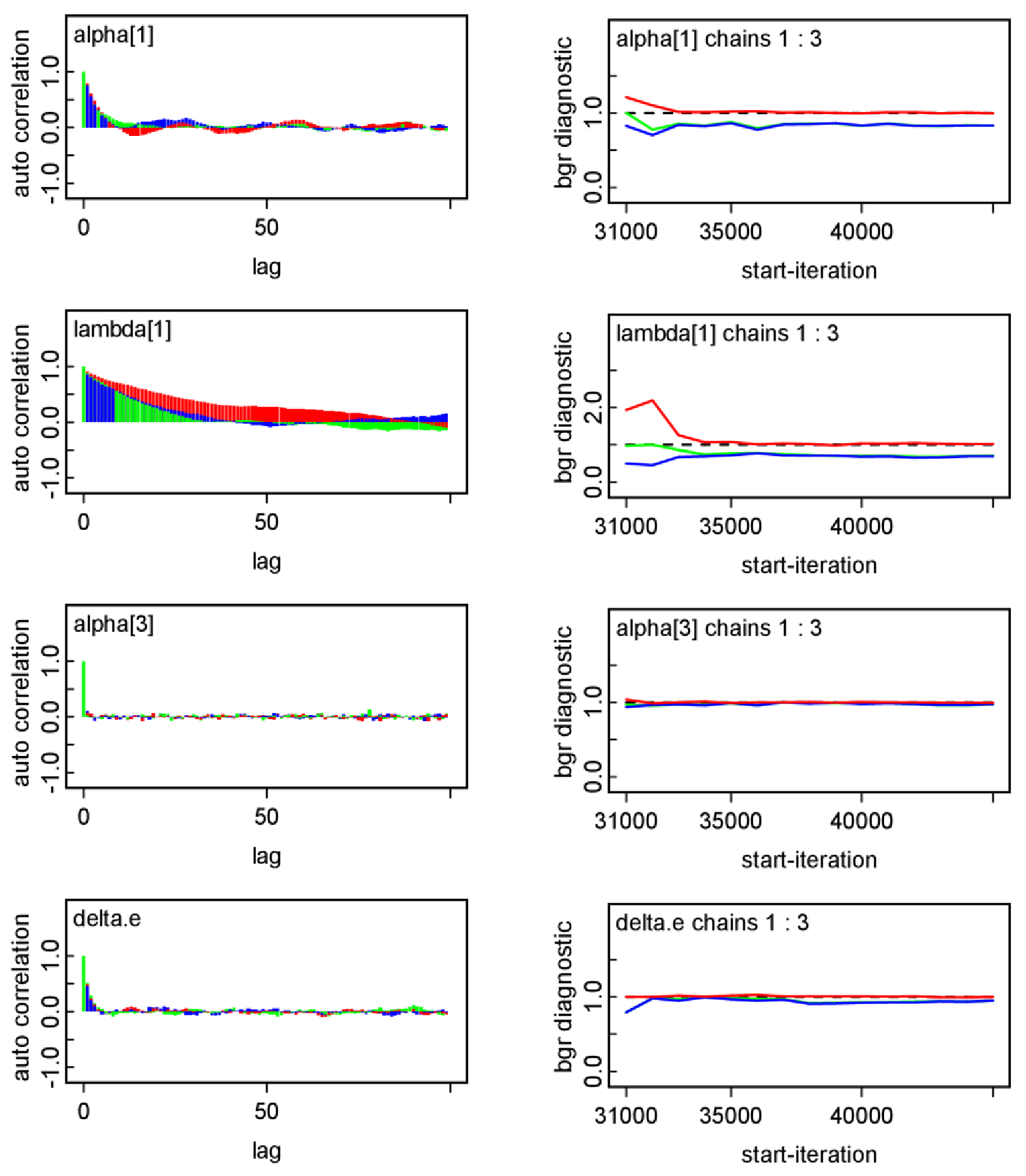


Figure A.2: Autocorrelation function plots (a) and BGR plots (b) of some representative parameters. The ACF plots indicate that there is low correlation of chains with their successive lags for each parameter. Additionally, the BGR plots reveal that the ratio of the BGR values come close to 1, further confirming convergence.

Table A.1 shows Geweke's test of convergence with the null hypothesis that the mean estimates from the early and latter parts of the MCMC are the same (chain convergence). None of the absolute values of Geweke's test statistics for the parameters exceeded the 95% critical value of 1.96, demonstrating strong evidence of convergence.

Table A.1: Results of the Geweke's test of convergence. The computed value of the test statistic for each parameter from the chosen model.

| alpha.1 | alpha.2 | alpha.3 | lambda.1 | lambda.2 | lambda.3 |
| --- | --- | --- | --- | --- | --- |
| 1.3068 | -0.9757 | -1.0072 | -0.6309 | -0.2138 | 0.7046 |
| delta.e | rho.e | sigma.e | sigma.phi11 | sigma.phi12 | sigma.phi13 |
| -0.4521 | -0.3862 | 0.1994 | -0.4874 | 0.3073 | 1.2526 |
| sigma.phi14 | sigma.phi21 | sigma.phi22 | sigma.phi23 | sigma.phi24 | sigma.phi31 |
| 0.8836 | 0.3073 | 1.0804 | -1.2299 | 0.2103 | 1.2526 |
| sigma.phi32 | sigma.phi33 | sigma.phi34 | sigma.phi41 | sigma.phi42 | sigma.phi43 |
| -1.2299 | -0.9808 | -0.6836 | 0.8836 | 0.2103 | -0.6836 |
| sigma.phi44 | deviance |  |  |  |  |
| 0.7158 | 0.1118 |  |  |  |  |

**Appendix B. Skew Distributions**

Symmetric distributions are not suitable for modelling some heavy-tailed longitudinal data. So, finding a more flexible method to well represent such data has received great attention recently. The most commonly used skew-elliptical distributions in the literature are multivariate skew-normal [11, 23] and multivariate skew-t [11, 19, 23] distributions. The model errors and/or random-effects in the longitudinal process submodel can be assumed to have skew-normal or skew-t distributions to make a robust inference.

**Multivariate Skew-normal Distribution**

Let $\boldsymbol{Y}={(Y_{1},Y_{2}, ..,Y_{m})}^{T}$ denotes an m-dimensional random vector, and follows a multivariate SN distribution, introduced by Sahu et al. [11], if its probability density function is given by

$$f\left( \boldsymbol{y}/{\boldsymbol{\mu,}\boldsymbol{\Sigma},\mathbf{Q}} \right)=2^{m}\left| \boldsymbol{B} \right|^{-1/2}\phi_{m}\left\{ \boldsymbol{B}^{-1/2}\left( \boldsymbol{y-\mu} \right) \right\}\Phi_{m}\left[ \left( I-\boldsymbol{Q}\boldsymbol{B}^{-1}\boldsymbol{Q} \right)^{-1/2}\boldsymbol{Q}\boldsymbol{B}^{-1}\left( \boldsymbol{y-\mu} \right) \right] (B.1)$$

Where $\boldsymbol{B}\boldsymbol{=\Sigma}+\mathbf{Q}^{2}$**;** $\phi_{m}(.)$ and $\Phi_{m}[.]$ represent the probability density and the cumulative distribution functions, respectively, of m-variate standard normal distribution $N_{m}(\boldsymbol{0},\boldsymbol{I}_{m})$; $\boldsymbol{\mu}$ is an m-dimensional location parameter; $\boldsymbol{\Sigma}$ is a $m\times m$ positive definite covariance matrix of $\boldsymbol{Y}$**,** and $\mathbf{Q}=diag(\delta_{1}, \delta_{2}, \ldots, \delta_{m})$ is an $m\times m$ diagonal skewness matrix. The distribution of Y can be denoted by ${SN}_{m}\left( \boldsymbol{\mu,}\boldsymbol{\Sigma},\mathbf{Q} \right)$. The mean and variance-covariance of $\boldsymbol{y\sim}{SN}_{m}\left( \boldsymbol{\mu,}\boldsymbol{\Sigma},\mathbf{Q} \right)$ are given by

$$\boldsymbol{E}\left( \boldsymbol{Y} \right)\boldsymbol{=\mu+}\sqrt{2/\pi}\boldsymbol{\delta}\mathrm{and} Cov\left( \boldsymbol{Y} \right)\boldsymbol{=}\boldsymbol{\Sigma+(}1\mathbf{-}2/\pi)\mathbf{Q}^{2} (B.2)$$

Where $\boldsymbol{\delta=}{(\delta_{1}, \delta_{2}, \ldots, \delta_{m})}^{T}$ is a skewness vector. In order to have a zero-mean random vector **Y**, it is assumed that the location vector $\boldsymbol{\mu=-}\sqrt{2/\pi}\boldsymbol{\delta}$. When $\boldsymbol{\Sigma}=diag({\sigma^{2}}_{1}, {\sigma^{2}}_{2}, \ldots, {\sigma^{2}}_{m})$, the pdf in equation (B.1) gives independent marginals and it reduces to

$$f\left( \boldsymbol{y}/{\boldsymbol{\mu,}\boldsymbol{\Sigma},\mathbf{Q}} \right)=\prod_{i=1}^{m} \left\{ \frac{2}{\sqrt{{\sigma^{2}}_{i}+{\delta^{2}}_{i}}}\phi\left( \frac{y_{i}-\mu_{i}}{\sqrt{{\sigma^{2}}_{i}+{\delta^{2}}_{i}}} \right)\Phi\left( \frac{\delta_{i}}{\sigma_{i}}\frac{y_{i}-\mu_{i}}{\sqrt{{\sigma^{2}}_{i}+{\delta^{2}}_{i}}} \right) \right\} (B.3)$$

Let $\boldsymbol{S}={(S_{1},S_{2}, ..,S_{m})}^{T}$ denotes a random vector. Then, a convenient hierarchical stochastic representation for a skew-normal (SN) distribution [11, 23] can be given as

$${\boldsymbol{y}/\boldsymbol{S}\boldsymbol{\sim}\boldsymbol{N}_{\boldsymbol{m}}\left( \boldsymbol{\mu+QS,}\boldsymbol{\Sigma} \right) \atop\boldsymbol{S\sim}\boldsymbol{N}_{\boldsymbol{m}}\left( \boldsymbol{0,}\boldsymbol{I}_{\boldsymbol{m}} \right)\boldsymbol{I(S>0)}} (B.4)$$

Notice that when **Q = 0**, the multivariate SN distribution reduces to the usual multivariate normal distribution.

**Multivariate Skew-t Distribution**

When robustness to potential outliers is a concern, as it is known the t-distribution is less peaked at the center and heavy-tailed and has been used as an alternative to the Gaussian distribution. Sahu et al. [11] developed a multivariate skew-t (ST) distribution by introducing skewness parameter. Let $\boldsymbol{y}={(Y_{1}, Y_{2}, \ldots, Y_{m} )}^{T}$ be an $m\times m$ random vector. Assume that $\boldsymbol{y}$ follows an m-variate skew-t distribution, $\boldsymbol{y\sim}{ST}_{m, \kappa}(\boldsymbol{\mu}, \sigma^{\boldsymbol{2}}\boldsymbol{I}_{m}\boldsymbol{,}\delta\boldsymbol{I}_{m})$ with probability density function [11]:

$$f\left( \boldsymbol{y}/{\boldsymbol{\mu,}\sigma^{\boldsymbol{2}},}\delta\mathbf{,}\kappa\right)=\frac{2^{m}}{{[\kappa\pi\left( \sigma^{\boldsymbol{2}}\boldsymbol{+}\delta^{\boldsymbol{2}} \right)]}^{m/2}} \frac{\Gamma\{\left( \kappa+m \right)/2\}}{\Gamma(\kappa/{2)}}\left\{ 1+\frac{\left( \boldsymbol{y}-\boldsymbol{\mu} \right)^{T}\left( \boldsymbol{y}-\boldsymbol{\mu} \right)}{\kappa\left( \sigma^{\boldsymbol{2}}\boldsymbol{+}\delta^{\boldsymbol{2}} \right)} \right\}^{{-(\kappa+m)}/2}\times T_{m,\kappa+m} \left[ \left\{ \frac{\kappa+{\left( \boldsymbol{y}-\boldsymbol{\mu} \right)^{T}\left[ \left( \sigma^{\boldsymbol{2}}\boldsymbol{+}\delta^{\boldsymbol{2}} \right)\boldsymbol{I}_{m} \right]}^{-1}\left( \boldsymbol{y}-\boldsymbol{\mu} \right)}{\kappa+m} \right\}^{-\frac{1}{2}}\frac{\delta}{\sigma}\frac{\left( \boldsymbol{y}-\boldsymbol{\mu} \right)}{\sqrt{\sigma^{\boldsymbol{2}}\boldsymbol{+}\delta^{\boldsymbol{2}}}} \right] (B.5)$$

Where $\Gamma(.)$ is a gamma function; $\delta$ denotes a skewness parameter; $\kappa$ denotes the degrees of freedom; $T_{m,\kappa+m}\left( . \right)$ is the cumulative density function of $t_{m,\kappa+m}(\boldsymbol{0}, \boldsymbol{I}_{m})$, and $\sigma^{\boldsymbol{2}}$ denotes a scale parameter (unknown).

The mean and variance-covariance of $\boldsymbol{y\sim}{ST}_{m, \kappa}(\boldsymbol{\mu}, \sigma^{\boldsymbol{2}}\boldsymbol{I}_{m}\boldsymbol{,}\delta\boldsymbol{I}_{m})\boldsymbol{,}$are given by

$$\boldsymbol{E}\left( \boldsymbol{y} \right)\boldsymbol{=\mu+}\sqrt{\kappa/\pi} \frac{\Gamma\{\left( \kappa-1 \right)/2\}}{\Gamma(\kappa/{2)}}\boldsymbol{\delta} (B.6)$$

$$Cov\left( \boldsymbol{y} \right)\boldsymbol{=}\left( \sigma^{\boldsymbol{2}}\boldsymbol{+}\delta^{\boldsymbol{2}} \right)\boldsymbol{I}_{m}\left( \frac{\kappa}{\kappa-2} \right)\boldsymbol{-}\frac{\kappa}{\pi}\left[ \frac{\Gamma\left\{ \left( \kappa-1 \right)/2 \right\}}{\Gamma(\kappa/{2)}} \right]^{\boldsymbol{2}}\delta^{\boldsymbol{2}}\boldsymbol{I}_{m}\boldsymbol{,}when \kappa>2 (B.7)$$

Where $\boldsymbol{\mu=}-g(\kappa)\boldsymbol{\delta}$. Let $\boldsymbol{S}$ and $u$ denote a random vector and a scaling weight, respectively. Then, following Sahu et al. [11], a hierarchical representation for a skew-t distribution can be given as

$${\boldsymbol{y}/\boldsymbol{S}\boldsymbol{\sim}\boldsymbol{N}_{\boldsymbol{m}}\left( \boldsymbol{\mu+Q[S-}g\left( \kappa\right)1_{m}]\boldsymbol{,}\frac{1}{u} \boldsymbol{\Sigma} \right)\boldsymbol{,} \atop\begin{aligned} \frac{\boldsymbol{S}}{u}\boldsymbol{\sim}\boldsymbol{N}_{\boldsymbol{m}}\left( \boldsymbol{0,}\frac{1}{u}\boldsymbol{I}_{\boldsymbol{m}} \right)\boldsymbol{I}\left( \boldsymbol{S>0} \right)\boldsymbol{,} \\ u/\kappa\boldsymbol{\sim}\boldsymbol{\Gamma}\boldsymbol{(}\kappa/2,\kappa/2\boldsymbol{)} \end{aligned}} (B.8)$$

Where the function $g\left( \kappa\right)=\sqrt{\kappa/m}\frac{\Gamma\left\{ \left( \kappa-1 \right)/2 \right\}}{\Gamma(\kappa/{2)}}$.

Lee and McLachlan [23] demonstrated a recent development of multivariate skew-normal and skew-t distribution mixtures. The densities and detailed specifications of both restricted and unrestricted multivariate mixture SN and ST distributions can be found in [23]. They also proposed the following more convenient hierarchical representation for a multivariate skew-t distribution:

$${\boldsymbol{y}/\boldsymbol{S}\boldsymbol{\sim}\boldsymbol{N}_{\boldsymbol{m}}\left( \boldsymbol{\mu+QS,}\frac{1}{u} \boldsymbol{\Sigma} \right)\boldsymbol{,} \atop\begin{aligned} \frac{\boldsymbol{S}}{u}\boldsymbol{\sim}\boldsymbol{N}_{\boldsymbol{m}}\left( \boldsymbol{0,}\frac{1}{u}\boldsymbol{I}_{\boldsymbol{m}} \right)\boldsymbol{I}\left( \boldsymbol{S>0} \right)\boldsymbol{,} \\ u/\kappa\boldsymbol{\sim}\boldsymbol{\Gamma}\boldsymbol{(}\kappa/2,\kappa/2\boldsymbol{)} \end{aligned}} (B.9)$$

Also notice that when $\kappa$ is large, the ${ST}_{m, \kappa}(.)$ reduces to the ${SN}_{m}(.)$, and when $\boldsymbol{Q=0}$, the ${ST}_{m, \kappa}(.)$ reduces to the usual $t_{m, \kappa}(.)$ distribution.
